# Largazole and Its Derivatives Selectively Inhibit Ubiquitin Activating Enzyme (E1)

**DOI:** 10.1371/journal.pone.0029208

**Published:** 2012-01-18

**Authors:** Dana Ungermannova, Seth J. Parker, Christopher G. Nasveschuk, Wei Wang, Bettina Quade, Gan Zhang, Robert D. Kuchta, Andrew J. Phillips, Xuedong Liu

**Affiliations:** 1 Department of Chemistry and Biochemistry, University of Colorado, Boulder, Colorado, United States of America; 2 Department of Chemistry, Yale University, New Haven, Connecticut, United States of America; Institute of Enzymology of the Hungarian Academy of Science, Hungary

## Abstract

Protein ubiquitination plays an important role in the regulation of almost every aspect of eukaryotic cellular function; therefore, its destabilization is often observed in most human diseases and cancers. Consequently, developing inhibitors of the ubiquitination system for the treatment of cancer has been a recent area of interest. Currently, only a few classes of compounds have been discovered to inhibit the ubiquitin-activating enzyme (E1) and only one class is relatively selective in E1 inhibition in cells. We now report that Largazole and its ester and ketone analogs selectively inhibit ubiquitin conjugation to p27^Kip1^ and TRF1 *in vitro*. The inhibitory activity of these small molecules on ubiquitin conjugation has been traced to their inhibition of the ubiquitin E1 enzyme. To further dissect the mechanism of E1 inhibition, we analyzed the effects of these inhibitors on each of the two steps of E1 activation. We show that Largazole and its derivatives specifically inhibit the adenylation step of the E1 reaction while having no effect on thioester bond formation between ubiquitin and E1. E1 inhibition appears to be specific to human E1 as Largazole ketone fails to inhibit the activation of Uba1p, a homolog of E1 in *Schizosaccharomyces pombe*. Moreover, Largazole analogs do not significantly inhibit SUMO E1. Thus, Largazole and select analogs are a novel class of ubiquitin E1 inhibitors and valuable tools for studying ubiquitination *in vitro*. This class of compounds could be further developed and potentially be a useful tool in cells.

## Introduction

In humans, protein ubiquitination is a dynamic process, depending on a tightly regulated balance between the activity of two ubiquitin-activating enzymes (E1s), approximately 40 ubiquitin-conjugating enzymes (E2s), and hundreds of ubiquitin ligases (E3s). Protein ubiquitination and subsequent degradation regulates almost every aspect of eukaryotic cellular function including cell cycle regulation, endocytosis, signal transduction, apoptosis, DNA damage repair, transcriptional regulation, and many others [1]. Hershko and coworkers discovered that ubiquitin covalently modified proteins prior to their degradation in rabbit reticulocyte lysates and characterized the reaction mechanism [2]. They first described the ubiquitin-activating enzyme, E1, that carries out the ATP-dependent activation of the C-terminal glycine residue of ubiquitin prior to ligation. In the first step of the E1 activation, the enzyme forms a complex with ubiquitin and ATP and catalyzes the adenylation of ubiquitin and successive release of pyrophosphate (PPi). During the second step, a thioester bond is formed between the C-terminus of ubiquitin and E1, subsequently releasing adenosine monophosphate (AMP). In the final step of E1 activation, additional ATP and ubiquitin are recruited to the adenylation site, generating a fully loaded E1 carrying two molecules of ubiquitin. The activated ubiquitin is then transferred to a cysteine in the active site of ubiquitin carrier protein E2, also via thiol ester linkage. Some E2 enzymes transfer ubiquitin to acceptor proteins directly, whereas other E2s require additional substrate binding proteins known as ubiquitin ligases or E3s [3], [4].Through this mechanism, ubiquitin is attached to proteins by isopeptide linkages between the C-terminal Gly76 of ubiquitin and the ε-amino groups of lysine residues present in substrate proteins. In addition, linkages between Lys48 of one ubiquitin and the C-terminal Gly76 of another ubiquitin ultimately form polyubiquitin chains [5]. Once polyubiquitinated, proteins are targeted by the 26S proteasome for degradation.

In many human cancers, the ubiquitination system is often destabilized. For example, the cyclin-dependent kinase inhibitor p27 is mainly regulated at the protein level and is excessively degraded in approximately 50% of all human cancers [6], [7]. Furthermore, expression of p27 is primarily controlled by polyubiquitination via the SCF^Skp2^ E3 ubiquitin ligase and subsequent proteasomal degradation [8]. The SCF^Skp2^ is a cullin-RING ligase (CRL), which is comprised of RING-box protein I (Rbx1), scaffold protein Cul1, linker protein Skp1, and F-box protein Skp2 [9]. In order for the ligase to function, Cul1 must first be covalently modified by NEDD8, an ubiquitin-like protein [10]–[12]. Therefore, an observed stabilization of p27 in cells could result from decreased polyubiquitination by inhibiting the neddylation of Cul1 or one of the enzymes required for ubiquitination.

Given that ubiquitination influences many cellular functions, malfunctions in the pathway play a role in the pathogenesis of human neurodegenerative disorders such as Parkinson's, Alzheimer's and Huntington's diseases, as well as cancer. Inhibiting components of the ubiquitination system seems to be an avenue of therapeutic development with clinical applications [13], [14]. For example, each E3 ligase targets a small number of proteins for ubiquitination, which makes it a potential target for highly specific inhibitors that have few side effects. There has, however, been little success in developing inhibitors of specific E3 ligases until recently [14], [15]. Also, proteasome-inhibiting compounds have been a target of interest and were originally developed as tools for probing its proteolytic function [16], [17]; however, these inhibitors were considered as possible cancer therapeutics after it was observed that they induced apoptosis in leukemic cell lines [18]–[20]. Although inhibiting the proteasome would nonspecifically inhibit the entire ubiquitination system, the proteasome inhibitor Bortezomib has fared surprisingly well in clinical trials and is now FDA approved for the treatment of relapsed and refractory myeloma and mantle cell lymphoma [19]. Therefore, inhibitory compounds of the ubiquitin system, whether they are specific or nonspecific, have the potential to be important therapeutics for the treatment of cancer.

In January 2008, the Leusch group at the University of Florida identified a natural product they named Largazole, which was isolated from cyanobacteria of the *Symploca* genus. They examined the compound for cytoxicity against cancer cells and observed remarkable antiproliferative activity in transformed mammary epithelial cells. In addition, they showed that Largazole preferentially targets cancer cells over normal cells, which makes this marine substance an important synthetic target as well as a potentially valuable cancer chemotherapeutic. Remarkably, the structure consists of several unusual features, such as a 16-membered macrocycle containing a 4-methylthiazoline fused to a thiazole ring and an octanoic thioester side chain, a unit rarely found in natural products. [21]. Also, Leusch and co-workers first reported the total synthesis of Largazole and determined that the molecular basis for its anticancer activity is HDAC inhibition [21], [24]. Numerous analogs of Largazole have been generated in efforts to understand the structure-activity relationship, and it has been determined that the thioester moiety is required for HDAC inhibition [21]–[32]. Here, we report *in vitro* mechanistic studies that reveal a potential role of Largazole as an antagonist of the ubiquitin-activating enzyme E1. In contrast to HDAC inhibition, ketone and ester analogs of Largazole can actively block the ligation of ubiquitin onto E1, indicating a differential mode of inhibitory activity since the formation of a thiol metabolite is indispensible for E1 inhibition. More explicitly, Largazole's presence negatively affected the formation of ubiquitin adenylate, which we monitored through nucleotide exchange assay.

## Materials and Methods

### Construction of Kip16, a GFP-p27 Expressing Cell Line

Mink lung epithelial cells expressing GFP-p27 were generated by retroviral-mediated gene transfer. pBabe-GFP-p27 amphotropic virus was made by cotransfecting pBabe-GFP-p27-Puro with pCL-Ampho in 293T cells. Viral supernatant was collected and used to infect mink lung epithelial cell line Mv 1 Lu (CCL-64) from ATCC in the presence of 8 µg/ml polybrene. Puromycin was added at 5 µg/ml and stable clones were selected. Each clone was subcultured and tested for GFP-p27 expression in the presence or absence of 10 µM MG132 (Calbiochem, Darmstadt, Germany) for 24 hours. Clones expressing high levels of GFP in the presence of MG132 but low or unndetectable GFP in its absence were expanded. Immunoblotting using an anti-p27 antibody (Santa Cruz Biotechnology, Santa Cruz, CA) was used to confirm the expression of the GFP-p27 fusion protein and stabilization of GFP-p27 upon MG132 treatment. One of the clones used for all subsequent studies was named Kip16.

### Largazole Treatment of Kip 16 cells

Total synthesis of Largazole and Largazole analogs is described in [24] within the supporting information (including copies of spectra of all compounds) and is available at http://pubs.acs.org. Kip16 cells were seeded into 96-well flat clear-bottomed plates at 40,000 cells/well in 100 µl medium and incubated overnight at 37°C in a humidified 5% CO2 atmosphere. Largazole was then added to final concentrations ranging from 1 µM to 1 nM in 300 µl of fresh medium. 0.3% DMSO and 1 µM of MG132 were used as negative and positive controls, respectively. After 24 hours of incubation, the medium was removed, the cells were washed twice with phosphate-buffered saline (PBS), and the cells were fixed with 4% paraformaldehyde in PBS for 15 minutes and stored at 4°C for microscopy evaluation. Cells were visualized with a GFP filter set using a 10× objective on an Eclipse TE2000-S (Nikon, Melville, NY) equipped with a Photometrics camera (Roper Scientific, Tucson, AZ).

### UBA1 and His-cdc34 Purification

Human ubiquitin E1 (UBA1) was expressed with an N-terminal GST tag fusion by means of recombinant baculovirus expression in Hi5 insect cells using the pFastBacHTA vector (Invitrogen, Carlsbad, CA). The cells were lysed by sonication in the presence of protease inhibitors in a buffer containing 200 mM NaCl, 50 mM Tris-HCl pH 7.5, 1% NP40, 1 mM DTT, and 1 mM EDTA. Cleared lysate was incubated with glutathione beads (Amersham, Sweden) for one hour at 4°C. After three washes with lysis buffer, untagged E1 was produced by thrombin cleavage. The protein solution was passed through a S200 gel filtration column (Amersham, Sweden), and UBA1 concentration and purity was evaluated by SDS-PAGE and Coomassie Blue gel staining. The purity was generally greater than 90% and purified UBA1 was aliquoted and stored at −80°C after quick freezing in liquid nitrogen. N-terminal hexahistidine (His)-tagged human Cdc34 was cloned into the pQE-30 vector (Qiagen, Valencia, CA) and expressed in *Eschericia coli*. His-cdc34 was purified by Ni-NTA chromatography followed by ion exchange and size-exclusion chromatography. The purity and concentration of His-cdc34 were determined by SDS-PAGE analysis.

### *In Vitro* Ubiquitination of p27 and Trf1

Mouse p27, cloned into pCS2, was translated *in vitro* in a reticulocyte lysate system (Promega, Madison, WI) in the presence of [^35^S]-labeled methionine. p27 was phosphorylated by purified recombinant Cdk2-CyclinE as outlined by Ungermannova et al [33]. 5 µl of the phosphorylation reaction was incubated with a ubiquitin mixture containing 100 nM UBA1, 200 nM His-cdc34, 100 nM SCF^Skp2^ E3 ligase complex, 50 nM Cks1, 10 µM ubiquitin (Sigma Aldrich, St. Louis, MO), 10 µM methylated ubiquitin (Boston Biochem, Cambridge, MA), 1 µl of energy regeneration system (noted as 20×ER and consisting of 10 mM ATP, 20 mM Tris-HCl pH 7.4, 100 mM MgCl_2_, 200 mM creatine phosphate, 2 mg/ml creatine phosphokinase, and 10% glycerol) , 1 µM ubiquitin aldehyde, and 1 µM MG132 in a total volume of 15 µl. The reaction was quenched after 30 minutes in a 30°C water bath by addition of 4× SDS sample buffer. The products of ubiquitination were resolved by SDS-PAGE, destained in a 45% methanol and 10% acetic acid solution in water, dried and exposed overnight to a phosphoimager screen, and scanned using a Typhoon scanner 9400 (GE Healthcare, Piscataway, NJ). *In vitro* Trf1 ubiquitination was carried out as described in Zeng et al [34]. Recombinant Trf1, labeled with [γ-^33^P]-ATP by CDK1-CyclinB, was incubated with 0.5 µM UBA1, 5 µM UbcH5a, 1 µM SCF^Fbx4^ E3 ligase complex, 5 µM ubiquitin, 100 µM methylated ubiquitin, 1 µM ubiquitin aldehyde, and 1 µl 20×ER for two hours at 30°C. Ubiquitinated Trf1 was analyzed by SDS-PAGE followed by autoradiography.

### E1/E2 Thioester Bond Formation Assay

40 nM ubiquitin E1 (UBA1) or 1 µM *S. pombe* E1 (Uba1p, gift from Chris Lima) or 0.5 µM human SUMO E1 (Boston Biochem, Cambridge, MA) were pre-incubated with 20×ER at 30°C for 5 minutes in thioester reaction buffer (20 mM Tris pH 7.6, 50 mM NaCl, and 10 mM MgCl_2_). After 5 minutes, 1 µM fluorescein-ubiquitin (Boston Biochem, U-590) was added to initiate attachment of ubiquitin. All components were allowed to react for another 5 minutes in a total volume of 5 µl. The reaction was stopped with 10 µl of SDS-PAGE loading buffer, minus DTT, and the proteins were resolved using 12% gels that were run on ice to prevent the reduction of E1-ubiquitin due to the heat generated during electrophoresis. Thioester bond formation was visualized by scanning the gel using Typhoon scanner 9400 (GE Healthcare) that was set to fluorescence mode (532 nM). When necessary 100 nM of E2 (Cdc34) was added after the E1 enzyme was pre-charged with ATP. Serially diluted Largazole and its analogs were incubated with the reagents as stated in the text. ImageJ was utilized to quantify the fluorescence signal, and the dose response curves were generated by nonlinear least regression analysis of data using Prism (GraphPad, San Diego, CA).

### [α-^32^P]-AMP: [α-^32^P]-ATP and [^32^P]-PPi:[γ-^32^P]-ATP Exchange Assays

The reaction mixture contained, in a final volume of 10 µl, 50 mM Tris-HCl pH 7.5, 150 mM NaCl, 10 mM MgCl_2_ (reaction assay buffer), 150 nM human ubiquitin E1 (UBA1), 100 µM ATP, 2 mM AMP, 1 µM [α-^32^P]- or [γ-^32^P]- ATP (Perkin Elmer, Waltham, MA), 500 µM PPi (sodium salt). A total of 5 µM ubiquitin was added to the mixture to initiate the ATP:AMP exchange. After incubation at 30°C for 10 minutes, the reactions were quenched with EDTA, and 0.5 µl aliquots of the reaction mixtures were spotted on Baker-flex® thin layer chromatography (TLC) polyethylenimine-modified cellulose plates (J.T. Baker, Phillipsburg, NJ) and developed in filtered 0.34 M potassium phosphate pH 7.0 for 20 minutes in a glass jar. The TLC plates were allowed to air dry for 10 minutes, covered in plastic wrap, and then exposed to a phosphoimager plate for 5–10 minutes. The separation of radiolabeled nucleotides was visualized using a Typhoon scanner 9400 (GE Healthcare, Piscataway, NJ).

## Results

### Largazole stabilizes GFP-p27 expression in Kip16 cells

**Figure 1:**
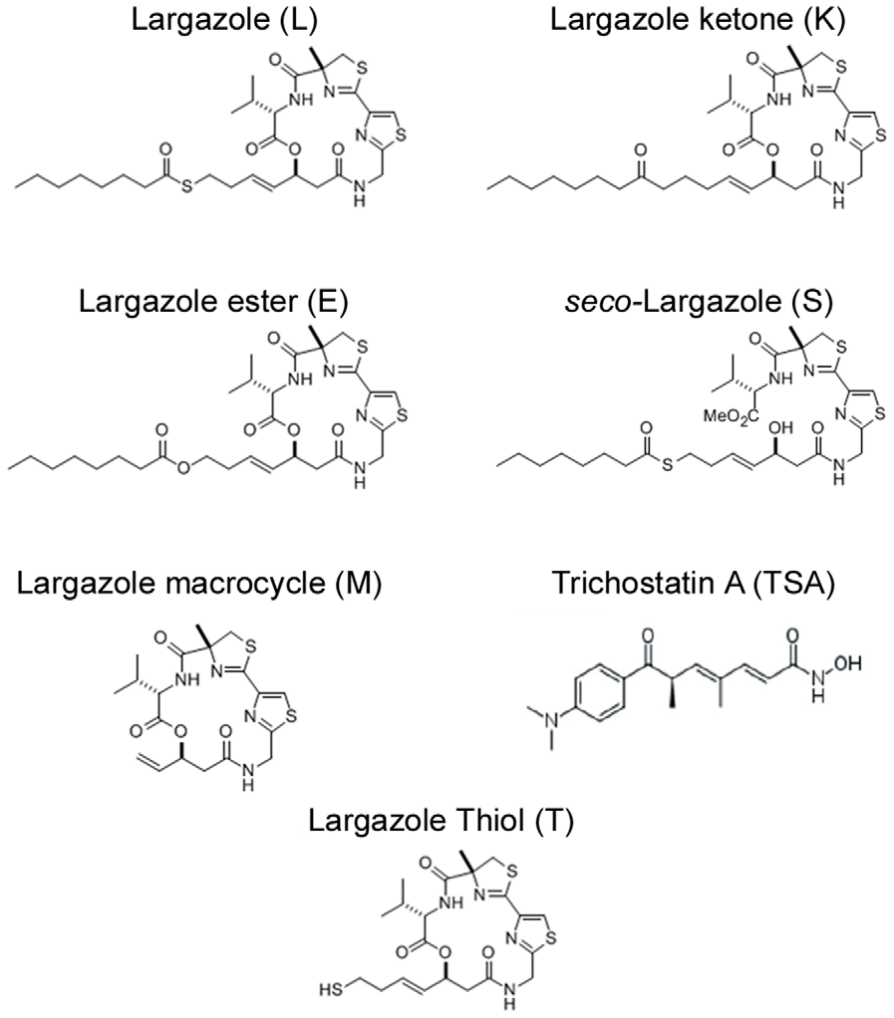
Chemical structures of Largazole, synthetic analogs, and Trichostatin A. Largazole (L) includes a substituted 4-methythiazoline linearly fused to a thiazole, a 3-hydroxy-7-mercaptohept-4-enoic acid, a thioester moiety, and a hydrocarbon tail. Analogs include a substituted ketone (K) and ester (E) in place of the thioester moiety, a macrocycle lacking the thioester moiety and hydrocarbon tail (M), an analog containing a macrocycle broken at carbon-3 of the enoic acid (S), and a thiol analog lacking the thioester moiety (T). Trichostatin A (TSA) contains a hydroxamic acid functional group.

**Figure 2:**
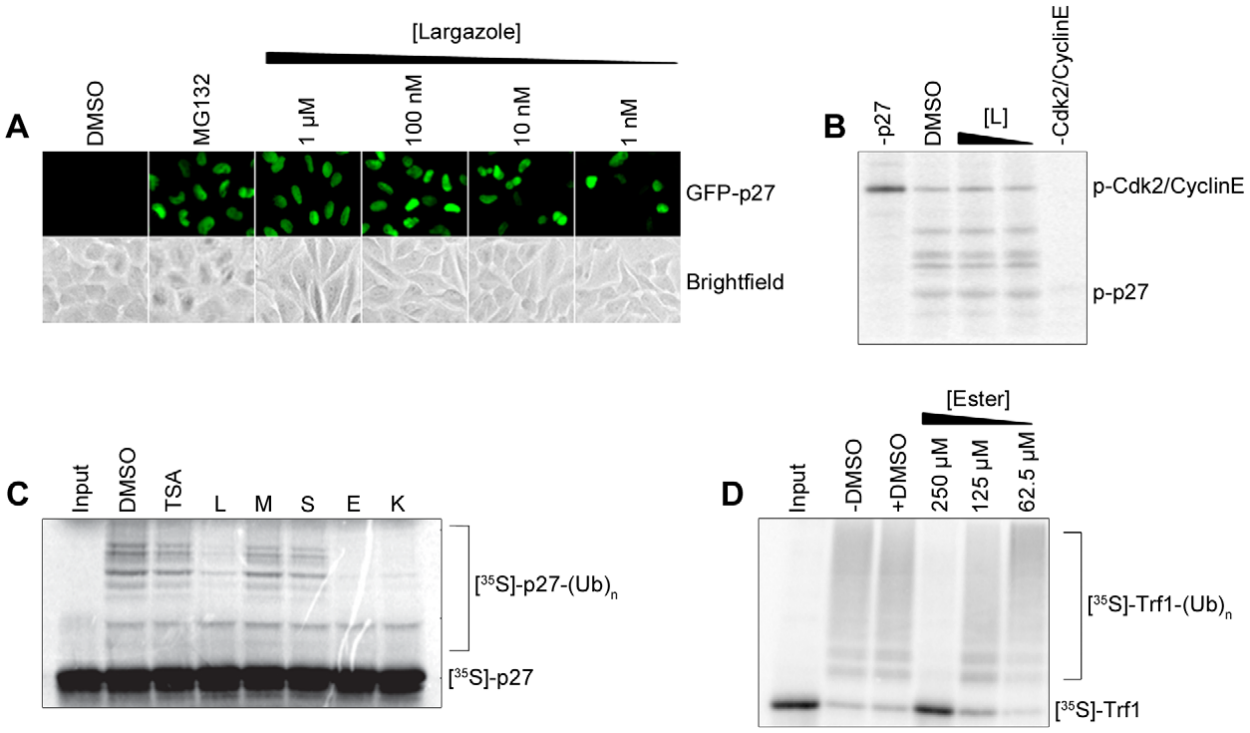
Largazole stabilizes p27 expression in Kip16 cells and inhibits p27 ubiquitination *in vitro.* (A) Fluorescent and corresponding bright-field images of Kip16 cells treated with varying concentrations of Largazole (L). L treatment induces the expression of GFP-p27 in a dose-dependent fashion. Addition of MG132 (1 µM) prevents the degradation of GFP-p27 via the ubiquitination and subsequent proteasomal degradation pathway. The vehicle control, DMSO, has no effect on the reporter protein stabilization. (B) L fails to inhibit the phosphorylation of p27 by the Cdk2/CyclinE complex compared to the positive control. L (250 µM, lane 3, and 125 µM, lane 4) was incubated with the Cdk2/CyclinE complex prior to the autophosphorylation of Cdk2/CyclinE step. Phosphorylated-p27 was identified by protein standard. (C) L, K, and E reduce polyubiquitinated forms of p27 while M and S have no inhibitory effects. Ubiquitin-activating enzyme E1 (100 nM), UBA1, was incubated with 100 µM of each compound prior to the reaction. (D) E reduces polyubiquitinated forms of Trf1 in a dose-dependent fashion. UBA1 (100 nM) was incubated with either DMSO or various concentrations of E ranging from 250 µM to 1 µM prior to the reaction.

A hallmark of many advanced cancers is an excessive degradation of the cyclin-dependent kinase inhibitor p27, which is chiefly directed by SCF^Skp2^-mediated ubiquitination. Hence, stabilization of p27 degradation represents a rational approach in cancer therapeutics. To identify small molecule inhibitors that can stabilize p27Kip1, we performed a screen of ∼3000 compounds from NCI DTP diversity set along with several natural products in our collection. For the cell-based screen, we generated a mink lung epithelial cell line (Kip16) stably expressing p27 that was cloned in frame with green fluorescent protein (GFP). The resulting N-terminal GFP-p27 fusion, detectable by fluorescence microscopy, was used to determine the levels of p27 expression upon treatment of cells with the compound libraries in 96-well format. Much to our surprise, the most potent hit that emerged from this screen was the natural compound Largazole (L) (Figure 1), which was first described by Luesch and coworkers [21] and subsequently synthesized in several laboratories including ours [21], [24]–[26], [29], [30], [32]. Largazole induced a robust and highly uniform upregulation of GFP-p27 at concentrations as low as 1 nM (Figure 2A). As expected, treatment with the proteasome inhibitor MG132 is highly effective in prevention of p27 degradation. We did not observe an increase in GFP-p27 expression upon treatment with the vehicle DMSO. This result suggests that Largazole can stabilize GFP-p27 expression in cultured cells.

### Largazole and select analogs inhibit the *in vitro* ubiquitination of p27 and Trf1

Before Largazole's function as an inhibitor of histone deacetylase was revealed, our initial investigation into the mechanism of this compound showed its ability to impede degradation of GFP-p27 in Kip 16 cells. One way to stabilize p27 is to block its ubiquitination. Hence we hypothesized that Largazole stabilizes p27 by inhibiting the ubiquitination pathway [7], [8]. One of the downsides of cell-based assays is that the effects observed may be attributed to the influence of multiple pathways. For example, inhibiting the proteasome, elevating transcription of p27, or inhibiting Cdk activity can also lead to an increase in p27 expression. To tease out the mechanism and action of Largazole on p27 stabilization, we decided to test the effect of Largazole on p27 ubiquitination in a fully reconstituted system *in vitro* [33], [35]. To test if Largazole affects p27 ubiquitination *in vitro*, we added Largazole to a p27 ubiquitin ligation reaction. As shown in Figure 2C, adding Largazole significantly reduced polyubiquitinated p27, suggesting that Largazole blocks p27 ubiquitination. Since Largazole is known to be a histone deacetylase inhibitor and has a thioester moiety that links an aliphatic chain to the core, we decided to test whether inhibition of p27 degradation can be linked to its histone deacetylase inhibitory activity. The structure-activity relationship for Largazole is relatively well understood [36]. Therefore we next tested a series of Largazole analogs (Figure 1) to study the effect of structure-activity relationship on p27 ubiquitination. To investigate this, Largazole ester (E), Largazole ketone (K), Largazole macrocycle (M), and *seco*-Largazole (S) were tested in an *in vitro* p27 ubiquitination assay (Figure 2C). We also added the HDAC inhibitor Trichostatin A (TSA), the structure of which can be found in Figure 1, to the assay to determine whether or not other HDAC inhibitors affect p27 ubiquitination. We observed that Largazole (L), Largazole ketone (K), and Largazole ester (E) inhibited the ligation of ubiquitin onto p27; however, the M and S analogs and TSA failed to inhibit the ubiquitination of p27 (Figure 2C). The fact that M had no inhibitory activity highlights the role of the octanoyl chain in hindering p27 polyubiquitination. *Seco-*Largazole (S) did not affect p27 ubiquitination, indicating the importance of the topology of the inhibitory compound. Furthermore, the result also suggests that the thioester moiety of Largazole is not required for inhibition, because the ketone and ester analogs were equally potent in blocking p27 ubiquitination. In addition, E1 inhibition is unrelated to HDAC inhibitor activity of Largazole as both ketone and ester fail to inhibit HDAC. Prior to ubiquitination, p27 is phosphorylated by the Cdk2-CyclinE complex. We carried out an *in vitro* p27 phosphorylation assay (as described in [33]) in the presence of either DMSO or Largazole in order to test whether or not the decrease in p27 ubiquitination was due to the inhibition of the Cdk2-CyclinE complex. We observed that Largazole does not inhibit the phosphorylation of p27 (Figure 2B); therefore, the inhibition of p27-ubiquitin conjugation is due to an inhibition of the ubiquitination process rather than phosphorylation step.

To study the specificity of largazole's inhibition, we set up Trf1 *in vitro* polyubiquitination in the presence of varying concentrations of largazole ester and found that E inhibited the ubiquitin attachment in a dose-dependent manner (Figure 2D). Since ubiquitination of both proteins was impeded, and given that both reactions require different factors to execute it (p27 has to be phosphorylated by CDK2-CyclinE while there is no requirement for Trf1 phosphate addition, E2 for p27 is Cdc34, while Trf1 needs Ubc5Ha, SCFSkp2 ligase works with p27 while Fbx4 is the substrate recognition subunit for Trf1) it was evocative that largazole compounds stall a step that is common to both polyubiquitination reactions.

### Largazole and ester/keto analogs inhibit ubiquitin E1 activation

**Figure 3:**
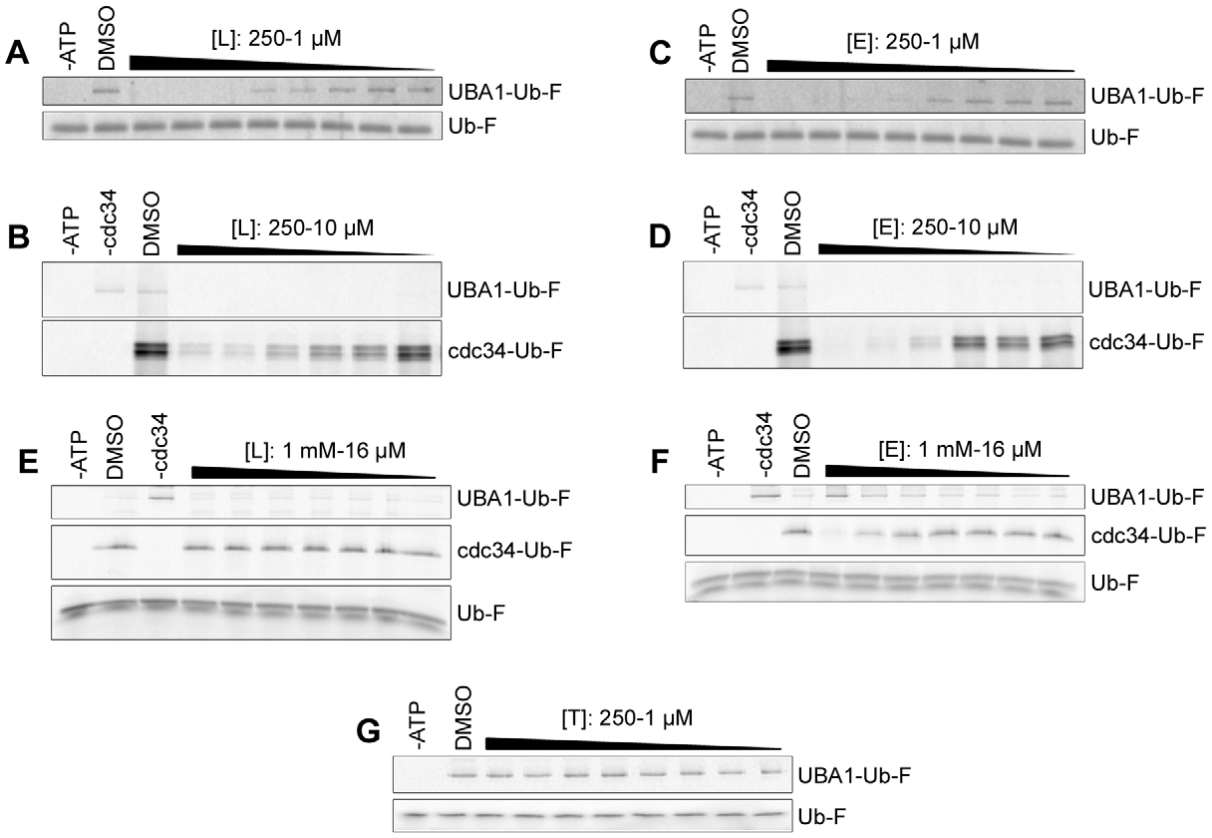
Largazole (L) and largazole ester (E) inhibit ubiquitin E1 in a dose dependent manner *in vitro*. (A,C) L and E inhibit transfer of ubiquitin onto E1 in a concentration-dependent manner. Thioester assay of E1 activity using fluorescein ubiquitin (Ub-F). Thioester bond formation between E1 and Ub-F is ATP-dependent (lane 2 vs. lane 1). In addition, DMSO has no effect on the formation of the thioester linkage as seen in lane 2 of both gels. 50 nM E1 was incubated with decreasing concentrations of L (A) or E (C) for 15 minutes at room temperature followed by addition of a cocktail containing ATP and Ub-F. After 5 minutes of incubation, the reactions were resolved by SDS-PAGE under non-reducing conditions. Ub-F was used to show equal loading. (B,D) Thioester assay of the ubiquitin transfer from E1 to E2 (Cdc34). Largazole or Largazole ester, when preincubated with 50 nM E1 for 15 minutes, inhibit the transfer of ubiquitin from E1 to Cdc34 in a concentration-dependent manner. (E) Largazole selectively inhibits the activity of E1 not E2. 50 nM E1 was pre-charged with ATP and then added to Cdc34 that was previously incubated with decreasing concentrations (1 mM–16 µM) of L in thioester reaction mixture. (F) Largazole ester inhibits E2 at high concentrations. Pre-charged E1 was added to reactions that contained Cdc34 pre-incubated with E ranging from 1 mM to 16 µM and resolved by SDS-PAGE under non-reducing conditions. Complete inhibition of ubiquitin transfer to E2 was observed at 1 mM of E, with only modest inhibition at 500 µM. (G) Largazole thiol (T) has no effect on transfer of ubiquitin onto E1. The reaction was carried out as described in A,C.

Since both p27 and Trf1 can be ubiquitinated in the presence of UBA1, we hypothesized that the inhibitory activity of Largazole is due to the deactivation of E1. To test this hypothesis, we incubated Largazole and Largazole ester with recombinant E1 prior to carrying out an *in vitro* thioester assay we described previously [37]. In addition, we tested the active thiol form of Largazole (T) for E1 inhibition. The presence of a fluorescence signal in the thioester assay suggests the formation of E1-ubiquitin adducts. The dose dependent decrease in fluorescence indicates that Largazole and Largazole ester inhibit the formation of E1-ubiquitin adducts (Fig. 3AC). The dose-response curves generated (data not shown) suggest an IC_50_ of approximately 29 µM and 25 µM for Largazole and Largazole ester, respectively. Interestingly, the active thiol form of Largazole (T) failed to inhibit E1 (Fig. 3G), suggesting again that the octanoyl residue is important for inhibition.

Activated ubiquitin is normally transferred to ubiquitin conjugating enzymes (E2). If E1 activity is inhibited, we expect to see that defects in E1 activation should impair the attachment of ubiquitin onto Cdc34 (E2). To further validate E1 inhibition, we included Cdc34, the E2 enzyme required for p27 ubiquitinaton, in the E1 reaction mixture. As shown in Figure 3EF, in the presence of ATP, fluorescent ubiquitin is transferred to Cdc34 indicated by the presence of a fluorescent Cdc34 band on the gel. Upon incubation with E1, Largazole or Largazole ester reduce the amount of ubiquitin molecules that are transferred from E1 to E2 in a dose-dependent fashion (Fig. 3BD).

The decreased ubiquitin transfer could be attributed to either E1 or E2 inhibition; therefore, we produced E1 precharged with ubiquitin by incubating ATP and fluorescent ubiquitin for 15 minutes at room temperature followed by the addition of Cdc34, which was preincubated with either Largazole or Largazole ester. If either compound inhibits the transfer of ubiquitin from E1 to E2, then we would observe a significant decrease in Cdc34 fluorescence regardless of the order we added the compounds. Interestingly, Largazole, preincubated with Cdc34, fails to inhibit the transfer of ubiquitin from precharged E1 at concentrations <1 mM (Fig. 3E). Furthermore, in a similar experiment, Largazole ester begins to inhibit the transfer of ubiquitin from precharged E1 to Cdc34 at concentrations around 500 µM (Fig. 3F), although this concentration is significantly above the IC_50_ of E1 inhibition. These results suggest that Largazole and Largazole ester exhibit selectivity towards ubiquitin E1. Also, this result suggests that either compound fails to promote the hydrolysis of ubiquitin thioesters on precharged E1.

### Largazole ketone inhibits the adenylation step of E1 activation

**Figure 4:**
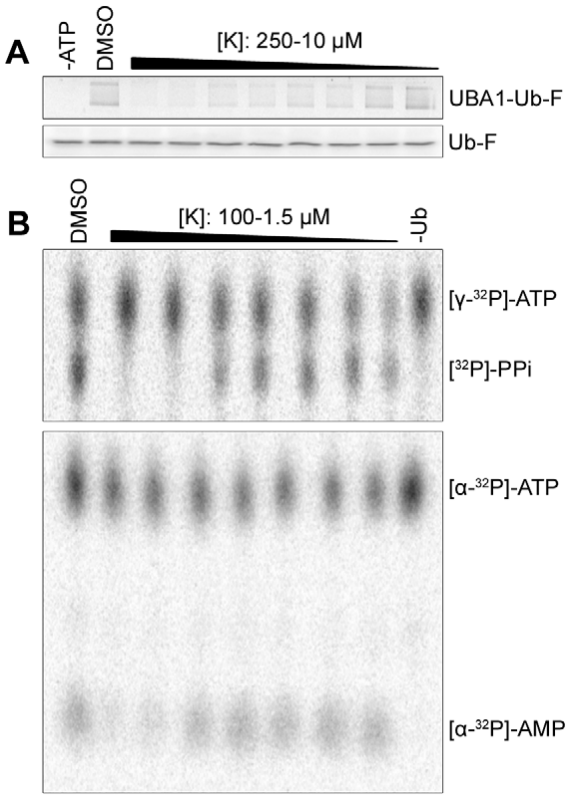
Largazole ketone inhibits the adenylation of the E1 ubiquitin-activating enzyme. (A) Largazole ketone inhibits ligation of ubiquitin onto E1 in a concentration-dependent fashion. Reduction of E1∼Ub adducts was determined by thioester assay utilizing fluorescein ubiquitin. (B) Largazole ketone inhibits the adenylation step in ubiquitin E1 activation in a concentration-dependent fashion. K was serially diluted (100 µM to 1.5 µM) and incubated with UBA1 (150 nM) at room temperature for five minutes. The thioester reaction mixture was mixed with ubiquitin to initiate the PPi:ATP exchange (middle panel) or AMP:ATP exchange (bottom panel) and added to the UBA1/K mixture. All reactions were halted with addition of EDTA after 10 minute incubation at 37°C, resolved using Cellulose PEI TLC plates, and analyzed using a phosphoimager.

E1 forms an ubiquitin–adenylate intermediate during the course of its catalytic cycle [3]. Thus the mechanism of ubiquitin E1 activation can be studied by assaying ATP:PPi and ATP:AMP exchanges [3]. Production of AMP in the [α-^32^P]-AMP:[ α-^32^P]-ATP exchange assay guarantees that a thioester bond is formed between E1 and ubiquitin, while the release of PPi, measured by the [^32^P]-PPi:[γ-^32^P]-ATP exchange assay, signals the formation of ubiquitin adenylate. To further dissect the mechanism of Largazole inhibition, two nucleotide exchange assays were carried out in the presence of Largazole derivatives. For these experiments we used Largazole ketone, which is similar to Largazole and Largazole ester. From the results shown in Figure 4, it is evident that the first two concentrations of Largazole ketone (100 and 50 µM) inhibit ubiquitination of E1 similarly and were also inhibitory in both types of exchange assays. The lack of a [^32^P]-PPi signal suggests that the adenylation step did not occur; consequently, ubiquitin could not be transferred to the active site cysteine to trigger the release of AMP. Both steps of the E1-catalyzed reactions can be measured by the AMP:ATP exchange assay. The lack of an [α-^32^P]-AMP signal further suggests that the adenylation step is inhibited by Largazole ketone. Thus Largazole or Largazole derivatives act on the first step of ubiquitin activation pathway by blocking the formation of ubiquitin-adenylate.

### Selectivity of Largazole ketone against SUMO E1 and Uba1p

**Figure 5:**
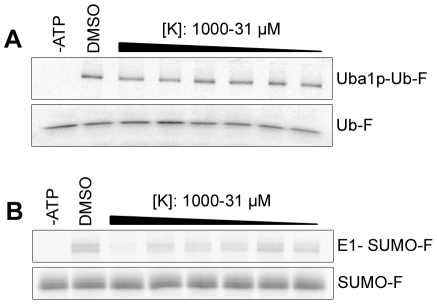
Investigation into the selectivity of Largazole ketone. (A) Largazole ketone (K) fails to inhibit the ligation of ubiquitin onto Uba1p, a homologue of UBA1 from *S. pombe*. Formation of Uba1p-ubiquitin adducts was determined by thioester assay utilizing fluorescein-ubiquitin. Uba1p (1.03 µM) was incubated with either DMSO or various concentrations of K serially diluted from 1000 µM to 31 µM. (B) K inhibits ligation of SUMO-1 onto human SUMO E1 in a concentration-dependent fashion. Reduction of E1-SUMO adducts was determined by thioester assay utilizing fluorescein-SUMO-1. hSUMO E1 (500 nM) was incubated with either DMSO or various concentrations of K serially diluted from 1000 µM to 31 µM.

In addition to ubiquitin, there exist several ubiquitin-like proteins that covalently modify other proteins. All of the ubiquitin-like proteins have activation pathways similar to ubiquitin [38]. In order to study the specificity of Largazole to the ubiquitin pathway, we incubated Largazole ketone with SUMO-activating E1 enzyme prior to carrying out a thioester assay. From the results in Figure 5B, we found that Largazole ketone is ineffective in inhibiting the formation of E1-SUMO adducts. From the dose-response curve generated from the SUMO E1 fluorescence results, the IC_50_ is approximately 450 µM as opposed to 30 µM for ubiquitin E1 (data not shown). Thus Largazole is relatively selective in perturbing ubiquitin E1 activation.

Ubiquitin and the ubiquitin E1 enzyme are highly conserved among eukaryotes [38]. Sequence analysis shows a 45% homology between the human ubiquitin-activating enzyme E1 (UBA1) and *S. pombe* E1 (ptr3/Uba1p) at the amino acid sequence level. To test whether Largazole ketone inhibits the *S. pombe* E1, we carried out a thioester assay using Largazole ketone and the ubiquitin E1 homologue in *S. pombe*, Uba1p. The results in Figure 5A suggest that Largazole ketone fails to inhibit the formation of E1-ubiquitin adducts at concentrations less than 1 mM. Taken together, these results suggest that Largazole and its derivative are highly selective in inhibiting the ubiquitin E1 enzyme.

## Discussion

In this study, we showed that Largazole and its analogs selectively inhibit ubiquitin E1 enzyme activity *in vitro*. Also, we demonstrated that the inhibitory activity of Largazole is independent of its inhibitory activity towards the histone deacetylase enzymes. Structure-activity relationship analysis shows that the thioester bond is not required for inhibition but the macrocycle core and aliphatic tail are indispensible. Largazole blocks ubiquitin activation at the adenylation step and without perturbing ubiquitin transfer from E1 to E2. Finally we show that Largazole inhibition of E1 is highly selective as it does not inhibit a highly related ubiquitin E1 enzyme from *S. pombe* and is almost twenty fold less effective in inhibiting the activation of SUMO E1. Taken together, our results reveal that Largazole represents a new class of ubiquitin E1 inhibitors.

We identified that Largazole caused a robust increase in GFP-p27 expression in Kip16 cells. This observation led us to further investigate the mechanism of GFP-p27 stabilization by Largazole. Using an *in vitro* ubiquitination assay, we were able to delineate the inhibitory point where Largazole acts on in the ubiquitination pathway, namely the E1 enzyme. However, there is a disconnect between the potency of E1 inhibition *in vitro* and GFP-p27 stabilization in cells. The EC_50_ of Largazole for GFP-p27 stabilization is in the low nM range, yet E1 inhibition is at ∼30 µM. This results suggests that the stabilization of GFP-p27 is unlikely caused by E1 inhibition, but is most likely a result of HDAC activity, which is known to block cell cycle progression and cause cell growth arrest. Consistent with this hypothesis, Largazole ketone and ester, two Largazole analogs that do not inhibit HDACs, do not increase GFP-p27 levels when Kip16 cells were treated (data not shown). However, other interpretations may account for the failure of Largazole ketone or ester to raise GFP-p27 by inhibiting E1 in cells. For example, we do not know if or how these compounds penetrate cells and how stable they are once they enter the cells. These investigations have to be undertaken before these analogs can be further developed for *in vivo* applications.

Panepophenanthrin, a natural compound derived from the mushroom strain *Panus rudis*, and Himeic acid A, derived from the marine fungus *Aspergillus*, are the first and second discovered inhibitors of the ubiquitin-activating enzyme E1, respectively [39], [40]. Both compounds were tested *in vitro* using recombinant E1; however, the cellular activity and mechanism were not determined [41]. PYR-41 and related pyrazones are another set of compounds that were discovered to inhibit ubiquitin E1 and the first set of E1 inhibitors described to enter cells and differentially kill transformed cells [42]. The IC_50_ of PYR-41 is around 5 µM, thus more potent than the compounds described here. However, the exact mechanism of PYR-41 inhibition is not known. Ub-AMSN represents a distinct class of protein based inhibitors of ubiquitin E1. Ub-AMSN contains a sulfamide group attached to the carboxyl terminus of ubiquitin as a nonhydrolyzable mimic of the phosphate group in the cognate Ub/Ubl-AMP adenylate intermediate. Thus, like Largazole analogs, it blocks the first step of E1 reaction [43], [44]. Unfortunately, Ub-AMSN cannot be used in cells as it cannot pass through the cell membrane. However, Ub-AMSN turns out be a very useful for probing the structure and biochemical mechanisms of E1 enzyme [44]. Therefore, Largazole and analogs could also be useful tools for probing ubiquitin function.

One of the most important questions to be answered is whether or not ubiquitin or ubiquitin-like E1 inhibitors are therapeutically relevant. Since only one ubiquitin E1 enzyme is responsible for a majority of protein ubiquitination in humans, inhibiting E1 will influence the degradation of proteins across several pathways and may lead to toxicity and, consequently, poor therapeutic efficacy. Bortezomib is the first FDA-approved proteasome inhibitor for the treatment of relapsed/refractory myeloma and mantle cell lymphoma. [19]. The proteasome, particularly the 26S proteasome, is the final step in ubiquitin-mediated protein degradation and regulates various pathways necessary for cellular function. The clinical success and efficacy of Bortezomib gives rise to the possibility that inhibitors of ubiquitin E1 will also share similar success. NEDD8 is a protein modifier that shares mechanistic and structural similarities to ubiquitin. Currently, the cullin family of proteins has been characterized as the target for NEDD8 conjugation [11]. MLN4924 is a potent and selective inhibitor of the NEDD8-activating enzyme (NAE) that exhibited potent cytotoxicity against several human tumor-derived cell lines [45]. Interestingly, MLN4924 shares a similar mechanism to Largazole analogs. MLN4924 reacts covalently with NEDD8 mimicking a NEDD8 adenylate that is incapable of driving the reaction forward, therefore, blocking the activity of NAE [46]. MLN4924 is currently undergoing phase I clinical trials in patients with lymphoma, multiple myeloma, or any form of nonhematologic malignancies. The *in vitro* and possible clinical success of the NAE inhibitor MLN4924 further supports the concept that E1 inhibitors are potential promising cancer therapeutics.

Our preliminary structure activity relationship studies suggest that the pro-drug form of Largazole including both the hydrocarbon tail and the macrocycle are essential for E1 inhibition. For Largazole analogs to be developed as potential antitumor drugs, additional analogs are needed to be synthesized in order to improve its potency toward ubiquitin E1. The most promising aspect of Largazole analogs as ubiquitin E1 inhibitors is the selectivity and specificity of Largazole. Largazole analogs not only display discrimination over related SUMO E1 enzyme but also remarkable selectivity in targeting human ubiquitin E1. Future structural studies would be helpful to understand how Largazole analogs inhibit E1, and insights gained from such studies may help to develop more specific inhibitors of E1. Experiments to test these hypotheses are currently underway.

## References

[1] Hershko A, Ciechanover A (1982). Mechanisms of intracellular protein breakdown.. Annu Rev Biochem.

[2] Hershko A, Ciechanover A, Rose IA (1979). Resolution of the ATP-dependent proteolytic system from reticulocytes: a component that interacts with ATP.. Proc Natl Acad Sci U S A.

[3] Haas AL, Warms JV, Hershko A, Rose IA (1982). Ubiquitin-activating enzyme. Mechanism and role in protein-ubiquitin conjugation.. J Biol Chem.

[4] Hershko A, Ciechanover A, Heller H, Haas AL, Rose IA (1980). Proposed role of ATP in protein breakdown: conjugation of protein with multiple chains of the polypeptide of ATP-dependent proteolysis.. Proc Natl Acad Sci U S A.

[5] Hershko A, Heller H (1985). Occurrence of a polyubiquitin structure in ubiquitin-protein conjugates.. Biochem Biophys Res Commun.

[6] Chu IM, Hengst L, Slingerland JM (2008). The Cdk inhibitor p27 in human cancer: prognostic potential and relevance to anticancer therapy.. Nat Rev Cancer.

[7] Pagano M, Tam SW, Theodoras AM, Beer-Romero P, Del Sal G (1995). Role of the ubiquitin-proteasome pathway in regulating abundance of the cyclin-dependent kinase inhibitor p27.. Science.

[8] Nakayama KI, Nakayama K (2006). Ubiquitin ligases: cell-cycle control and cancer.. Nat Rev Cancer.

[9] Petroski MD, Deshaies RJ (2005). Function and regulation of cullin-RING ubiquitin ligases.. Nat Rev Mol Cell Biol.

[10] Chiba T, Tanaka K (2004). Cullin-based ubiquitin ligase and its control by NEDD8-conjugating system.. Curr Protein Pept Sci.

[11] Pan ZQ, Kentsis A, Dias DC, Yamoah K, Wu K (2004). Nedd8 on cullin: building an expressway to protein destruction.. Oncogene.

[12] Podust VN, Brownell JE, Gladysheva TB, Luo RS, Wang C (2000). A Nedd8 conjugation pathway is essential for proteolytic targeting of p27Kip1 by ubiquitination.. Proc Natl Acad Sci U S A.

[13] Nalepa G, Rolfe M, Harper JW (2006). Drug discovery in the ubiquitin-proteasome system.. Nat Rev Drug Discov.

[14] Hoeller D, Dikic I (2009). Targeting the ubiquitin system in cancer therapy.. Nature.

[15] Garber K (2005). Missing the target: ubiquitin ligase drugs stall.. J Natl Cancer Inst.

[16] Vinitsky A, Cardozo C, Sepp-Lorenzino L, Michaud C, Orlowski M (1994). Inhibition of the proteolytic activity of the multicatalytic proteinase complex (proteasome) by substrate-related peptidyl aldehydes.. J Biol Chem.

[17] Vinitsky A, Michaud C, Powers JC, Orlowski M (1992). Inhibition of the chymotrypsin-like activity of the pituitary multicatalytic proteinase complex.. Biochemistry.

[18] Imajoh-Ohmi S, Kawaguchi T, Sugiyama S, Tanaka K, Omura S (1995). Lactacystin, a specific inhibitor of the proteasome, induces apoptosis in human monoblast U937 cells.. Biochem Biophys Res Commun.

[19] Orlowski RZ, Kuhn DJ (2008). Proteasome inhibitors in cancer therapy: lessons from the first decade.. Clin Cancer Res.

[20] Shinohara K, Tomioka M, Nakano H, Tone S, Ito H (1996). Apoptosis induction resulting from proteasome inhibition.. Biochem J.

[21] Taori K, Paul VJ, Luesch H (2008). Structure and activity of largazole, a potent antiproliferative agent from the Floridian marine cyanobacterium Symploca sp.. J Am Chem Soc.

[22] Bowers AA, West N, Newkirk TL, Troutman-Youngman AE, Schreiber SL (2009). Synthesis and histone deacetylase inhibitory activity of largazole analogs: alteration of the zinc-binding domain and macrocyclic scaffold.. Org Lett.

[23] Chen F, Gao AH, Li J, Nan FJ (2009). Synthesis and biological evaluation of c7-demethyl largazole analogues.. Chem Med Chem.

[24] Nasveschuk CG, Ungermannova D, Liu X, Phillips AJ (2008). A concise total synthesis of largazole, solution structure, and some preliminary structure activity relationships.. Org Lett.

[25] Ghosh AK, Kulkarni S (2008). Enantioselective total synthesis of (+)-largazole, a potent inhibitor of histone deacetylase.. Org Lett.

[26] Seiser T, Kamena F, Cramer N (2008). Synthesis and biological activity of largazole and derivatives.. Angew Chem Int Ed Engl.

[27] Souto JA, Vaz E, Lepore I, Poppler AC, Franci G (2010). Synthesis and biological characterization of the histone deacetylase inhibitor largazole and C7- modified analogues.. J Med Chem.

[28] Wang B, Huang PH, Chen CS, Forsyth CJ (2011). Total Syntheses of the Histone Deacetylase Inhibitors Largazole and 2-epi-Largazole: Application of N-Heterocyclic Carbene Mediated Acylations in Complex Molecule Synthesis.. J Org Chem.

[29] Ying Y, Liu Y, Byeon SR, Kim H, Luesch H (2008). Synthesis and activity of largazole analogues with linker and macrocycle modification.. Org Lett.

[30] Ying Y, Taori K, Kim H, Hong J, Luesch H (2008). Total synthesis and molecular target of largazole, a histone deacetylase inhibitor.. J Am Chem Soc.

[31] Zeng X, Yin B, Hu Z, Liao C, Liu J (2010). Total synthesis and biological evaluation of largazole and derivatives with promising selectivity for cancers cells.. Org Lett.

[32] Bowers A, West N, Taunton J, Schreiber SL, Bradner JE (2008). Total synthesis and biological mode of action of largazole: a potent class I histone deacetylase inhibitor.. J Am Chem Soc.

[33] Ungermannova D, Gao Y, Liu X (2005). Ubiquitination of p27Kip1 requires physical interaction with cyclin E and probable phosphate recognition by SKP2.. J Biol Chem.

[34] Zeng Z, Wang W, Yang Y, Chen Y, Yang X (2010). Structural basis of selective ubiquitination of TRF1 by SCFFbx4.. Dev Cell.

[35] Wang W, Ungermannova D, Chen L, Liu X (2004). Molecular and biochemical characterization of the Skp2-Cks1 binding interface.. J Biol Chem.

[36] Newkirk TL, Bowers AA, Williams RM (2009). Discovery, biological activity, synthesis and potential therapeutic utility of naturally occurring histone deacetylase inhibitors.. Nat Prod Rep.

[37] Knuesel M, Cheung HT, Hamady M, Barthel KK, Liu X (2005). A method of mapping protein sumoylation sites by mass spectrometry using a modified small ubiquitin-like modifier 1 (SUMO-1) and a computational program.. Mol Cell Proteomics.

[38] Kerscher O, Felberbaum R, Hochstrasser M (2006). Modification of proteins by ubiquitin and ubiquitin-like proteins.. Annu Rev Cell Dev Biol.

[39] Sekizawa R, Ikeno S, Nakamura H, Naganawa H, Matsui S (2002). Panepophenanthrin, from a mushroom strain, a novel inhibitor of the ubiquitin-activating enzyme.. J Nat Prod.

[40] Tsukamoto S, Hirota H, Imachi M, Fujimuro M, Onuki H (2005). Himeic acid A: a new ubiquitin-activating enzyme inhibitor isolated from a marine-derived fungus, Aspergillus sp.. Bioorg Med Chem Lett.

[41] Eldridge AG, O'Brien T (2010). Therapeutic strategies within the ubiquitin proteasome system.. Cell Death Differ.

[42] Yang Y, Kitagaki J, Dai RM, Tsai YC, Lorick KL (2007). Inhibitors of ubiquitin-activating enzyme (E1), a new class of potential cancer therapeutics.. Cancer Res.

[43] Olsen SK, Capili AD, Lu X, Tan DS, Lima CD (2010). Active site remodelling accompanies thioester bond formation in the SUMO E1.. Nature.

[44] Lu X, Olsen SK, Capili AD, Cisar JS, Lima CD (2010). Designed semisynthetic protein inhibitors of Ub/Ubl E1 activating enzymes.. J Am Chem Soc.

[45] Soucy TA, Smith PG, Milhollen MA, Berger AJ, Gavin JM (2009). An inhibitor of NEDD8-activating enzyme as a new approach to treat cancer.. Nature.

[46] Brownell JE, Sintchak MD, Gavin JM, Liao H, Bruzzese FJ (2010). Substrate-assisted inhibition of ubiquitin-like protein-activating enzymes: the NEDD8 E1 inhibitor MLN4924 forms a NEDD8-AMP mimetic in situ.. Mol Cell.

